# Norepinephrine May Exacerbate Septic Acute Kidney Injury: A Narrative Review

**DOI:** 10.3390/jcm12041373

**Published:** 2023-02-09

**Authors:** Lou’i Al-Husinat, Alameen Alsabbah, Amer Abu Hmaid, Razan Athamneh, Majd Adwan, Mohammad N. Hourani, Seif Almakhadmeh, Zaid Jehad Al Modanat, Mohammed I. A Ismail, Giustino Varrassi

**Affiliations:** 1 Department of Clinical Medical Sciences, Faculty of Medicine, Yarmouk University, Irbid 21163, Jordan; 2 Faculty of Medicine, Yarmouk University, Irbid 21163, Jordan; 3 Department of General Surgery and Anesthesia, Faculty of Medicine, Mutah University, Al-Karak 61710, Jordan; 4 Paolo Procacci Foundation, 00193 Roma, Italy

**Keywords:** norepinephrine, sepsis, septic shock, septic acute kidney injury, vasopressin, vasopressor

## Abstract

Sepsis, the most serious complication of infection, occurs when a cascade of potentially life-threatening inflammatory responses is triggered. Potentially life-threatening septic shock is a complication of sepsis that occurs when hemodynamic instability occurs. Septic shock may cause organ failure, most commonly involving the kidneys. The pathophysiology and hemodynamic mechanisms of acute kidney injury in the case of sepsis or septic shock remain to be elucidated, but previous studies have suggested multiple possible mechanisms or the interplay of multiple mechanisms. Norepinephrine is used as the first-line vasopressor in the management of septic shock. Studies have reported different hemodynamic effects of norepinephrine on renal circulation, with some suggesting that it could possibly exacerbate acute kidney injury caused by septic shock. This narrative review briefly covers the updates on sepsis and septic shock regarding definitions, statistics, diagnosis, and management, with an explanation of the putative pathophysiological mechanisms and hemodynamic changes, as well as updated evidence. Sepsis-associated acute kidney injury remains a major burden on the healthcare system. This review aims to improve the real-world clinical understanding of the possible adverse outcomes of norepinephrine use in sepsis-associated acute kidney injury.

## 1. Plain Language Summary

Sepsis is a common and potentially life-threatening complication of infection. When sepsis leads to hemodynamic instability, septic shock occurs, which can result in septic acute kidney injury (S-AKI). S-AKI must be treated quickly. A patient with S-AKI experiences low blood pressure and decreased circulation to the kidneys. Treatment involves administering fluids to the patient and providing a vasopressor agent to improve circulation. The first-line vasopressor in use is norepinephrine, although other agents are also used. Recent studies have suggested that norepinephrine may result in adverse outcomes. In particular, norepinephrine increases the glomerular filtration rate, which increases sodium delivery into the tubules of the renal medulla. This sodium requires oxygen for reabsorption, which means that norepinephrine results in a decrease in oxygen to the kidneys. While vasopressin does improve microcirculation, these improvements do not always affect the microcirculation of the kidneys. This narrative review describes the pathophysiology of sepsis, septic shock, S-AKI, and the role of norepinephrine and other vasopressors in treating S-AKI.

## 2. Key Points

1. Septic-AKI (S-AKI) is clinically distinct from AKI without sepsis.
2. Microcirculatory abnormalities can occur in S-AKI due to endothelial injury and shedding of the glycocalyx, which can result in reduced blood flow velocity.
3. Norepinephrine is recommended as the first-line treatment for S-AKI. It increases the glomerular filtration rate, increases sodium filtration and reabsorption, boosts renal blood flow, and has no effect on urine flow or renal vascular resistance. However, norepinephrine can reduce medullary tissue oxygen by half.
4. Although 20% of cardiac output is delivered to the renal system, the renal medulla is vulnerable to hypoxia, likely due to vascular congestion.
5. In head-to-head clinical studies of norepinephrine vs. vasopressin, there was no clear superiority for norepinephrine, and in some studies, vasopressin had lower mortality. In meta-analyses, vasopressin did not confer a clear-cut mortality benefit over standard care.

## 3. Introduction

Sepsis is a life-threatening condition that results from a profoundly dysregulated response to infection. It can lead to organ failure distant from the primary site of the infection, particularly in the kidneys [1]. Sepsis has been associated with a hyperinflammatory response and concomitant immune suppression [2]. Almost 50 million cases of incident sepsis have been reported around the world, resulting in approximately 11 million deaths. From a global perspective, sepsis accounts for 19.7% of all deaths, with the greatest burden in Sub-Saharan Africa, Oceania, and Asia [3]. The global incidence of sepsis is greatest in children and neonates, but there are scant population-based data on this subject; it has been estimated to be 3 million neonates and 1.2 million children with sepsis [4].

An expert consensus definition of septic shock defined it as a subset of sepsis associated with circulatory, cellular, and metabolic abnormalities, such that septic shock has a greater mortality risk than sepsis alone [5]. Sepsis may be thought of as an infection with organ dysfunction [6]. Septic shock is diagnosed in adults when vasopressor treatment is required to maintain a mean blood pressure of 65 mmHg or higher and where there is a serum lactate level > 2 mmol/L after fluid resuscitation [5]. Vasopressors are typically administered to maintain mean arterial pressure ≥ 65 mmHg [1].

Sepsis can cause acute kidney injury (AKI) [7], but the rate of AKI in critically ill patients varies. Although up to 60% of patients with sepsis will develop AKI [8], there is limited information about the epidemiology of sepsis-associated AKI (S-AKI), which has emerged as a common complication in hospitalized patients [9]. S-AKI patients may experience any of several disease trajectories, including renal recovery, survival with diminished kidney function, or death [7]. The outcome depends greatly on the severity of the kidney injury. A 97-center international study of 1032 intensive care unit (ICU) patients in their first week in the ICU found that 57.3% had AKI, and the severity of the AKI was related to mortality [10]. Inpatients treated for AKI remain at risk for adverse kidney-related outcomes for six months [11]. Although one out of three septic patients will develop S-AKI [7], the underlying processes and pathogenesis of S-AKI remain to be elucidated.

Septic shock is a risk factor for S-AKI (odds ratio 2.88, 60.5%), but there are other risk factors, including type 2 diabetes mellitus, abdominal infection, use of vasopressors (odds ratio 2.95, 64.5%), vasoactive drugs (odds ratio 3.85, 63.22%), mechanical ventilation, history of smoking, cardiovascular diseases, and liver diseases [12]. It has been suggested that in S-AKI, the defects in the initial two days of the condition are more functional than structural in nature (i.e., abnormal microvasculature and tubular stress are more evident in the first 48 h than aberrant histopathology) [13]. As intrarenal perfusion is redistributed during sepsis, renal hypoxia may result, which may cause S-AKI [13]. Hypoxia is particularly damaging to the renal medulla and can lead to oxidative stress, initiating an inflammatory cascade, which leads to spiraling cellular injury [14]. Thus, tissue perfusion is a primary goal in managing S-AKI that can be accomplished with rapid infusion of intravenous (IV) fluids under ultrasound guidance [15]. If hypotension persists after fluid resuscitation, a vasopressor may be administered, typically norepinephrine [16]; if hypotension persists after norepinephrine administration, vasopressin may be used as well [17]. While vasopressors, such as norepinephrine, are widely used for blood pressure regulation and organ perfusion, paradoxically, norepinephrine does not appear to confer such benefits in AKI [13]. Thus, the optimal therapy for S-AKI is not known, nor are the underlying mechanisms of this potentially life-threatening condition. The goal of our narrative review was to evaluate the literature with the goal of developing a better understanding of S-AKI and how it might inform prescribing choices.

## 4. Methods

Using the PubMed database, the keywords “septic acute kidney injury” were searched in November 2022, with results limited to the past 10 years (2012–2022) and including only clinical trials and randomized clinical trials published in English with associated data available. This yielded 63 results. These were then sorted to include those that reviewed the use of norepinephrine or vasopressin in the setting of S-AKI, which yielded one result. A search for “septic acute kidney injury norepinephrine” yielded 24 results, three of which were relevant. A search for “septic acute kidney injury vasopressin” yielded three results, two of which were duplicates from other searches; the remaining one was not relevant. The next search was for “septic acute kidney injury”, but it was limited to meta-analyses in the past 10 years in English; this yielded 28 results. We excluded meta-analyses regarding renal replacement therapy or not directly related to norepinephrine or vasopressin; this yielded four results, which are summarized in Table 1. Using the same keywords “septic acute kidney injury”, but limiting the search to systematic reviews in the past 10 years, yielded 33 results. In this search, all relevant results had been found in previous searches. Using the same criteria, we searched Scopus (14 documents, all included in the previous findings) and Web of Science (18 documents, all included in the previous findings). In addition, we reviewed the bibliographies of these studies. The objective of our narrative review was to provide a thorough background on S-AKI and then review these relevant studies on the use of norepinephrine in such cases.

## 5. Results

Sepsis can be defined as any dysregulated response to an infection. It can trigger septic shock, an inflammatory cascade that may involve a cytokine storm [22], along with circulatory system abnormalities and metabolic and cellular derangements [12]. Septic shock can lead to AKI, which may be life threatening or have long-term adverse sequelae [8]. S-AKI is clinically distinct from AKI without sepsis. A high proportion, if not the majority, of critically ill patients develop S-AKI [23], which carries with it high morbidity and mortality rates [12]. Sepsis and AKI form a vicious cycle, as sepsis is one of the main drivers of critical illness [23].

The host’s response to any pathogen depends in part on the virulence of the micro-organism. The type of pathogen in sepsis helps determine the course of the infection and the outcome. The predominant organisms associated with sepsis are *Staphylococcus aureus* (20.5%), *Pseudomonas spp.* (19.9%), *Enterobacteriaceae* (16.0%), and fungi (19%) [24]. In a meta-analysis of 510 studies, gram-negative bacteremia was more closely associated with mortality than gram-positive bacteremia [25]. While the organism and site of infection may play a role in determining negative outcomes, approximately one-third of patients with severe sepsis do not have a positive blood culture [26]. Genetic factors play a role in sepsis susceptibility and poor outcomes [24]. Comorbidities may also play a role, as sepsis contributes to about 30% of all in-hospital cancer deaths [24]. The increasing incidence of sepsis and S-AKI may be due, in part, to the emergence of progressively more multidrug-resistant pathogens, longer lifespans, more urinary catheterizations, and older patients living with chronic diseases and “managed cancer” [27,28,29,30].

Sepsis leads to numerous complications, including acute respiratory distress syndrome (ARDS) [31] and disseminated intravascular coagulopathy (DIC), which can lead to microthrombosis, ischemic limb injury, and death [32]. Sepsis may also cause delirium, along with psychological or cognitive problems [33]. One of the most commonly reported complications of sepsis is AKI, which increases mortality risk [34].

### 5.1. Diagnostic Challenges in S-AKI

The Acute Dialysis Quality Initiative group published a landmark consensus definition for AKI in adults known as RIFLE, for the five stages of AKI: risk, injury, failure, loss, and end-stage disease [35]. AKI was characterized by a creatinine increase of ≥50% from baseline and/or a decrease in glomerular filtration rate (GFR) of ≥25% and/or decreased urinary output below a level of 0.5 mL/kg/h over at least six hours. RIFLE stated that the ≥50% increase in creatinine level occurred or could be reasonably presumed to have occurred over ≤ 7 days [35]. The AKI Network (AKIN) amended RIFLE by omitting the last two stages (loss and end-stage renal disease) and establishing the following stages: stage 1: risk, stage 2: injury, and stage 3: failure [36]. Inflammatory biomarkers, such as procalcitonin (PCT), C-reactive protein (CRP), and interleukin 18 (IL-18), are under consideration as potential diagnostic tools for S-AKI [37]. An increase of 0.3 mg/dL in creatinine over 48 h was part of stage 1, and the GFR criteria were removed [36]. Kidney Disease: Improving Global Outcomes (KDIGO) synthesized RIFLE and AKIN with some modifications [38].

The traditional biomarkers for AKI were urinary output, urinary indices, tubular enzymes, and cystatin C [39]. Biomarkers that indicate damage include neutrophil gelatinase-associated lipocalin (NGAL), kidney injury molecule-1 (KIM-1), interleukin-18 (IL-18), and liver-type fatty acid-binding proteins [37,40,41,42,43]. However, the evidence for some of these biomarkers is suggestive rather than conclusive, and some remain controversial. Biomarkers specific to AKI in the setting of sepsis (S-AKI) are emerging and include differentially expressed genes [44]. The identification of hub genes for AKI and hub genes for septic shock resulted in datasets that allowed for the identification of potential targets for future research. The genes under consideration associated with S-AKI are VMP1, SLP1, PTX3, TIMP1, OLFM4, LCN2, and S100A9 [44].

### 5.2. Managing S-AKI

Sepsis is managed with fluid resuscitation and early administration of antimicrobial therapy [41]. The CLASSIC multicenter feasibility trial explored a protocol that restricted the volume of resuscitation fluid to 151 septic shock patients and found a benefit in reducing fluids [45]. The CLASSIC study pointed out the potential risks of fluid overload, defined as total input minus total output divided by initial body weight. Adverse events were associated with fluid overloads over approximately 10% [46,47]. However, the results of this feasibility study remain controversial [46]. The 2016 Surviving Sepsis Campaign (SSC) suggests a fixed dose of crystalloid fluid (30 mL/kg) for the first three hours from diagnosis [48], but others recommend individualizing fluids for each patient based on their clinical status, starting with an infusion of 10 mL/kg for the first 30–60 min [49]. Both the 2016 SSC guidelines [50] and the 2018 SSC bundle [51] recommend early use of vasopressors, such as norepinephrine and vasopressin, in severely hypotensive septic patients, titrating the doses up until a mean arterial pressure (MAP) of at least 65 mmHg is achieved. Close clinical monitoring and regular checking of vital signs are necessary; invasive blood pressure monitoring may be considered in cases in which vasopressors are used [50].

The role of renal replacement therapy in S-AKI is beyond the scope of this narrative review. While there is no expert consensus as to the appropriate time to commence dialysis, there is growing consensus that early initiation of renal replacement therapy in appropriate patients may be associated with improved outcomes [52].

### 5.3. The Pathophysiology of S-AKI

AKI is characterized by a rapidly declining glomerular filtration rate and the inability of the renal system to regulate fluid and electrolyte homeostasis [53]. The three main underlying causes of AKI are prerenal, postrenal, and intrinsic. Prerenal kidney injury is characterized by an abrupt reduction in blood flow to the kidney, resulting in kidney dysfunction, although the kidney itself is not damaged [54]. On the other hand, postrenal kidney injury occurs when the urinary tract below the kidneys is obstructed in some way, causing waste to build up in the kidneys [55]. S-AKI is caused by molecular patterns from pathogenic bacteria that are released from damaged cells; downstream effects are glomerular dysfunction, peritubular endothelial damage, downregulation of tubular resorption, apoptosis, and destruction of organelles from damaged cells [56]. Although S-AKI has been modeled as being secondary to renal ischemia, new findings from animal studies suggest that renal blood flow may paradoxically increase as renal vascular resistance decreases, so that S-AKI could occur in renal hyperemia. Ischemia need not be present for the loss of glomerular filtration [57]. Thus, S-AKI is a complex condition. Sepsis-induced renal microvascular dysfunction can occur, such as vasoconstriction, capillary leak syndrome, endothelial dysfunction, microthrombi, and others, leading to S-AKI [58]. A crucial development in S-AKI occurs when increased renal vascular resistance causes changes in the microcirculation of the renal cortex and/or renal medulla, despite normal or even elevated renal blood flow [58].

Historically, the pathophysiology of S-AKI was considered to be hypoperfusion and secondary tubular epithelial cell death because the most frequent causes of AKI were associated with decreased renal blood flow and ischemia [7]. Advances in postmortem and in vitro studies have revealed that hypoperfusion and/or ischemia are not the sole causes of S-AKI. It has been determined that hypoperfusion need not be involved in the pathogenesis of S-AKI, and S-AKI may be multifactorial in nature, with potential contributions from the inflammatory process, metabolic reprogramming, and dysregulated microcirculation [7].

**Figure 1:**
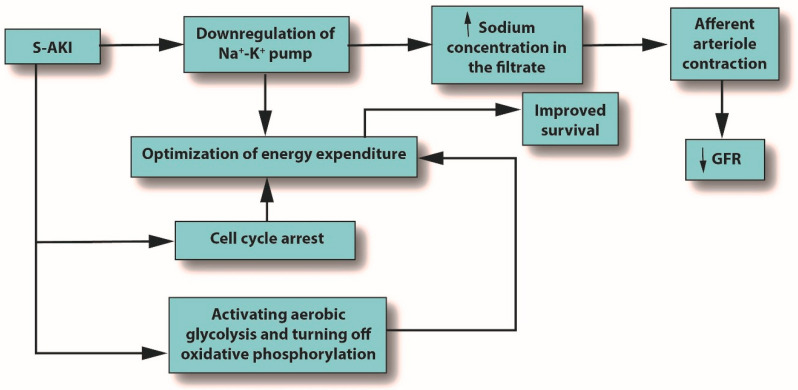
The outcomes of S-AKI. Note that the glomerular filtration rate is reduced only due to the influx of sodium produced by downregulation of the sodium/potassium pump; it is not an initiator. Abbreviations: GFR, glomerular filtration rate; S-AKI, septic acute kidney injury.

Inflammation is the body’s primary response to invading pathogens, and a dysregulated or aberrant inflammatory response may be caused by organ dysfunction [50]. In sepsis, the invading pathogen releases inflammatory mediators called pathogen-associated molecular patterns (PAMPs) into the circulation. In response, damaged cells release damage-associated molecular patterns (DAMPs). Both PAMPs and DAMPs interact with pattern recognition receptors (PRRs), which are part of the body’s innate immune system [59]. PRR signaling leads to pro-inflammatory responses aimed at destroying or at least subduing the infectious assault [59,60]. For example, Toll-like receptors (TLRs) are a type of PRR that are expressed in renal tubular epithelial cells [61]. When DAMPs and PAMPs interact with TLRs in tubular epithelial cells, an imbalance in the asymmetric pro- and anti-inflammatory actions can cause a pathological increase in oxidative stress, culminating in the release of reactive oxygen species (ROS), which can damage host tissues and cause organ dysfunction [62]. This release of ROS can trigger inflammation in the kidney, which may be distant from the main site of the infection [7]. In an effort to protect itself from free radicals, which can cause necrosis, protective proteins, such as NGAL and hepcidin, are released [63,64]. Macrophages associated with H-ferritin contribute to the hepcidin-associated protective anti-inflammatory effects [65]. See Figure 1.

**Figure 2:**
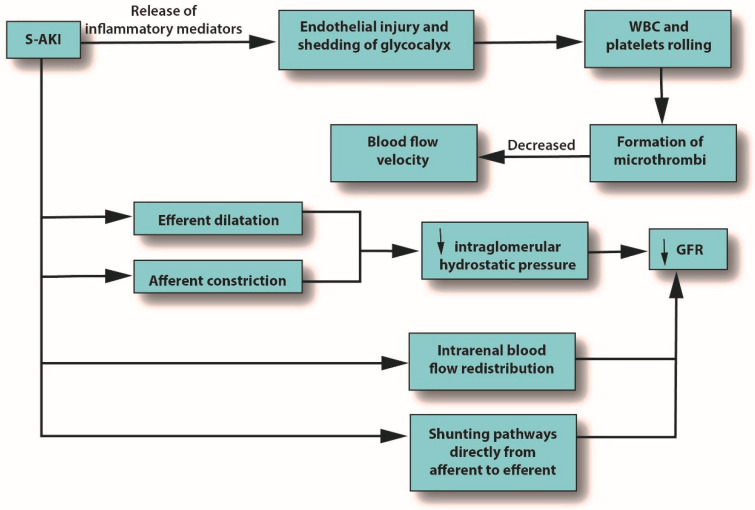
The role of pro-inflammatory mediators in S-AKI. Abbreviations: GFR, glomerular filtration rate; S-AKI, septic acute kidney injury; WBC, white blood cells.

Severe microcirculatory dysfunction can occur in S-AKI, regardless of macrohemodynamic stability [7]. Alterations in the microcirculation can be caused by endothelial injury and shedding of the glycocalyx, leading to the potential formation of microthrombi due to increasing levels of white blood cells (WBCs) and platelet rolling, which eventually decrease blood flow velocity. Microcirculatory changes can also reduce GFR by several mechanisms: reduced intraglomerular hydrostatic pressure due to afferent arteriole constriction, dilatation of the efferent arteriole, intrarenal blood flow redistribution away from the medulla, and the emergence of capillaries going from afferent to efferent arterioles without passing through the glomerulus [7]. See Figure 2.

**Figure 3:**
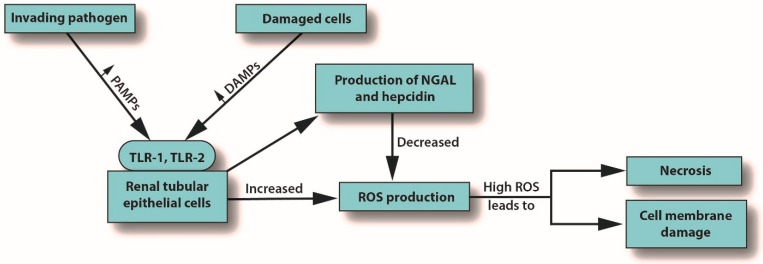
How S-AKI can lead to cell membrane damage and necrosis. Abbreviations: DAMPs, damaged-associated molecular patterns; NGAL, neutrophil gelatinase-associated lipocalin; PAMPs, pathogen-associated molecular patterns; ROS, reactive oxygen species; TLR, Toll-like receptor.

Metabolic reprogramming plays a major role in the development of S-AKI and has multiple mechanisms, including cell cycle arrest, downregulation of ion transporters, and switching from aerobic glycolysis to oxidative phosphorylation. All of these mechanisms seek to optimize energy expenditure [7]. See Figure 3.

The inflammation caused by sepsis alone can reduce GFR, but in fact, many other mechanisms can cause or contribute to decreased GFR [66]. When pro-inflammatory mediators reach the glomerulus, the mesangial cells within Bowman’s capsule contract, narrowing their already narrow pores to the point that filtration is hindered [67]. In AKI or S-AKI, renal cells optimize their energy expenditure in response to these stresses, sometimes using the pathophysiologic system of cellular hibernation [68,69]. In cellular hibernation, the sodium/potassium ATPase pump is downregulated; normally, this pump is responsible for about 80% of renal oxygen consumption [70]. This causes the *macula densa* to detect an increase in sodium concentration in the filtrate, triggering contraction of the afferent arteriole, which, in turn, will decrease GFR [71].

Of course, GFR may be adversely affected by any of several mechanisms that essentially cause an imbalance between the afferent and efferent arterioles, even in the presence of increased renal blood flow to the kidneys [72]. This reduced GFR was found to be caused by vasodilation of the renal arteries, which lends credence to the notion that overall renal function depends on the complex interplay between systemic hemodynamics and local renal microcirculation rather than the isolated effects of each one separately [73].

### 5.4. Septic Shock

Septic shock is the most severe complication of sepsis and is potentially life threatening [74]. The pathophysiology may be described as sequential intracellular events being launched by pathogens in immune cells, the epithelium, the endothelium, and, overall, in the neuroendocrine system [74]. To combat the pathogens, pro-inflammatory mediators are launched, which, in turn, causes the release of anti-inflammatory mediators to control the inflammatory response. The inflammatory response damages tissues, while the anti-inflammatory events cause leukocyte reprogramming and immune system alterations. This complicated process can happen in a very short window of time [74], and many interventions are intended to provide prompt symptomatic relief [75]. Septic shock is associated with abrupt and profound circulatory compromise, typically addressed with a vasopressor to maintain a mean arterial pressure of at least 65 mmHg and a serum lactate level greater than 2 mmol/L (>18 mg/dL) in the absence of hypovolemia [5].

### 5.5. Norepinephrine in S-AKI

Synthesized mainly in the locus coeruleus (LC) of the brain, norepinephrine is a catecholamine with diverse neuromodulatory effects, including regulation of the central nervous system (CNS). There are three main receptors for norepinephrine: α1-adrenoreceptors (α1Rs), α2-adrenoreceptors (α2Rs), and β-adrenoreceptors (BRs) [76]. Norepinephrine attenuates the release of pro-inflammatory mediators [77,78] and is recommended as a vasopressor in sepsis for its ability to regulate blood pressure and vascular resistance [79]. The cardiovascular effects of norepinephrine include an increase in the intracellular calcium concentration of smooth muscles and inotropic effects on the myocardium due to the effects of norepinephrine on α-adrenergic receptors [80]. Norepinephrine has many effects. It increases GFR, sodium filtration and reabsorption, renal blood flow, and oxygen delivery and consumption; however, it has no effect on renal vascular resistance, filtration fraction, urine flow, or renal oxygen extraction [81]. Norepinephrine reduces medullary tissue oxygen tension by 50% and decreases medullary perfusion. Since norepinephrine increases GFR, it increases sodium delivery into the medullary tubules [14,82]. This sodium requires oxygen for reabsorption, which leads to relative medullary hypoxia [82]. It has been maintained that this decreased renal medullary perfusion and inflammatory response contribute to AKI in septic patients because renal vasoconstriction decreases renal blood flow, which can lead to hypoperfusion and/or ischemia; however, this view has been challenged or perhaps better stated expanded [83]. Instead, it has been proposed that a dysfunctional renal tubular system activates microvascular shunting, causing a tubule-glomerular vicious circle, which, in turn, results in an inflammatory response and coagulation [83,84]. Renal hypoperfusion caused by sepsis likely does more than just reduce glomerular filtration, as it seems to trigger both macro- and microcirculatory deficits as well [85]. The renal medulla is particularly susceptible to hypoxia, despite the fact that approximately 20% of cardiac output is delivered to the renal system [86]. A putative explanation is that the outer portion of the medulla is vulnerable to vascular congestion, which can prolong hypoxia and lead to ischemia [86].

Catecholamine infusions in critically ill patients have been shown to trigger renal vasoconstriction and loss of renal function. Adjunctive to catecholamines is the use of vasopressin and/or norepinephrine. The current method of increasing renal perfusion in the setting of sepsis or S-AKI is the administration of vasopressin or norepinephrine to increase renal perfusion by restoring the microcirculatory balance [87]. One drawback of this approach is that macrocirculatory improvements do not always correspond to optimization of the microcirculation when inflammation or infection are present [88].

Terlipressin is a vasopressin analog with pronounced vasoconstrictive effects on the efferent arterioles and minimal effects on the afferent arterioles [89]. In a pilot study of 22 septic shock ICU patients, the patients were randomized to receive terlipressin or standard care [87]. The sonographic signal intensity peak at 24 h was 60.5 ± 8.6 dB among the terlipressin patients compared to 52.4 ± 70 dB in the control group (*p* = 0.04). The terlipressin patients had a lower time to peak, slower heart rates, and higher cardiac stroke volumes at 24 h after enrolling in the study vs. the control patients. There was no significant difference in urinary output at 24 h [87]. It should be noted that terlipressin was more effective than placebo in treating patients with hepatorenal syndrome type 1 [90]. AKI in patients suffering from acute liver failure was also more effectively treated with early infusion of terlipressin compared to noradrenaline, which conferred a mortality benefit [91]. In a study of 678 geriatric patients undergoing major gastrointestinal surgery, the incidence of postoperative AKI was 10.9%, but it was significantly lower in patients with mean arterial pressure controls set to a range of 80–95 mmHg compared to two other groups, where the mean arterial pressure was set to 65–79 mmHg or 96–110 mmHg. The patients in the highest mean arterial pressure group used more norepinephrine, phenylephrine, and nitroglycerin than the other two groups with lower range values [92].

Vasopressor administration to critically ill patients, including those with kidney dysfunction, is generally guided by blood pressure monitoring, with mean arterial pressure targets of 65 mmHG, although this number is somewhat higher for geriatric, hypertensive, or coronary artery disease patients [16]. To evaluate whether the use of vasopressors in older patients was associated with greater mortality, a randomized trial reduced the vasopressor exposure of older patients (≥65 years) by setting the mean arterial pressure target lower, at 60–65 mmHG, but this did not reduce the 90-day mortality rate [93]. In this study, 48% of the patients had septic shock, and 22% had no sepsis at all. Most patients (60%) received norepinephrine only, although other drugs (metaraminol and phenylephrine) were used in some patients [93].

In a double-blind single-center study of 76 critically patients with AKI receiving vasopressor therapy, a high continuous venovenous hemofiltration cutoff had no effect on the duration of vasopressor therapy or mortality compared to standard venovenous hemofiltration [94].

In a randomized study of 300 patients with risk factors for AKI undergoing cardiac bypass surgery, norepinephrine was used to maintain mean arterial pressure at either 75–85 mmHg (high group) or 50–60 mmHg (control group). Defining AKI as an increase in serum creatinine of 30% or more, both groups had the same proportion of AKI cases (17% vs. 17%, *p* = 1). Statistically similar were the lengths of stay, 28-day mortality, and six-month mortality [95].

Norepinephrine was directly compared with vasopressin in a study of 778 adults with septic shock [96]. Via a blinded infusion, patients received vasopressin (0.01–0.03 U/min) or norepinephrine (5–15 µg/min) along with open-label administration of vasopressors. Upon entry into the study, the overall proportion of patients with kidney injuries was 59.6% (*n* = 464). Vasopressin was associated with a lower rate of progression to kidney failure (20.8% vs. 39.6%, *p* = 0.03) and a lower need for renal replacement therapy (17.0% vs. 37.7%, *p* = 0.02). Furthermore, the vasopressin group had a lower mortality rate than the norepinephrine group [96].

In a randomized, double-blind study of 778 septic shock patients receiving at least 5 µg/min norepinephrine infusion, patients were randomized to receive either 0.01–0.03 U/min of vasopressin or 5–15 µg/min of norepinephrine. The 28-day and 90-day mortality rates were similar between groups, as were the rates of serious adverse events. However, in the subpopulation of patients with less severe septic shock, vasopressin was associated with a lower mortality rate than norepinephrine at 28 days (26.5% vs. 35.7%, *p* = 0.05) [97].

## 6. Discussion

The results of this research have made clear that this topic would deserve attention. At the moment, the literature is offering a few studies. Systematic reviews and meta-analyses have been offered to provide a broad picture of the role of vasopressin and/or norepinephrine in treating S-AKI. See Table 1. The first of them is focused on the differences between vasopressin and norepinephrine, with uncertain results [21]. Another one has investigated the possibility to reduce the administration of vasoactive drugs in elderly critically ill people [20]. The results were very uncertain and discussable. A meta-analysis including the results obtained on 788 patients treated with early administration of vasopressin (6 h septic shock) did not show any advantage with the comparator group, treated with the usual protocol [18]. A further study was addressed to identify the procedures used in intensive care, when patients with AKI where treated [19]. The authors identified 18 different protocols in studies suggesting a significant effect on mortality. The deep analysis of their investigation revealed an incredible discordance between participants stated beliefs and actual practice. 

Despite the increasing incidence of sepsis and the morbidity and mortality burden it presents, it is surprising how little research has been dedicated to the condition and its consequences [98]. Norepinephrine appears to have potentially adverse effects on renal function, which may increase the risk of AKI in patients with sepsis. As a result, it appears that other vasopressors may be better suited as first-line treatment, or they should be supplemented with other substances [99,100].

The clinical implications are profound. S-AKI is prevalent in critically ill patients and poses treatment challenges. Prompt fluid resuscitation and treatment are needed, but this is a severe condition with high morbidity and mortality rates. The role of norepinephrine is being superseded by vasopressin, although an expert consensus for a specific treatment protocol is lacking. More research into this difficult and potentially deadly condition is urgently needed. Since there are logistic and ethical challenges to conducting clinical trials in critically ill patients, it is likely that the best evidence for guiding treatment will come from a better understanding of the pathophysiology of S-AKI.

This study has several important limitations. It is a narrative review rather than a systematic review or meta-analysis. Many studies cited in this paper did not consider S-AKI or septic shock as variables. The doses of norepinephrine varied among the studies. Many animal studies, which we did not include, have been conducted to research S-AKI, and the results of some animal studies do not always align with human studies. Finally, the studies we reviewed sometimes had patient populations with specific comorbidities, such as diabetes mellitus, or demographic differences, such as geriatric patients. Thus, these findings are not generalizable to all patients.

## 7. Conclusions

Sepsis is prevalent and associated with morbidity and mortality. Despite this prevalence, understanding of the pathophysiology of S-AKI is limited. Norepinephrine is recommended as the first-line treatment, but a growing body of evidence suggests that vasopressin may be a better choice. Further study is needed.

## Figures and Tables

**Table 1:** Meta-analyses of clinical trials about the role of vasopressin and/or norepinephrine in the treatment of S-AKI.

Studies and Patients	Agents	Findings	Safety	Comments
Huang 2021 [18] 5 studies 788 patients	Vasopressin infusion within 6 h of developing septic shock vs. standard care	Short-term mortality was similar between groups	Similar rates of new-onset arrhythmias	No difference between groups in ICU length of stay
Landoni 2013 [19] 18 interventions	Observational of 18 different interventions	Reduced mortality; vasopressin in septic shock, terlipressin for hepatorenal syndrome type 1		15/18 interventions reduced mortality
Mouncey 2021 [20] 65 CCUs 2463 patients ≥ 65 years	Vasopressin vs. standard care	Vasopressin did not reduce 90-day mortality over standard care		Not blinded
Nagendrahn 2019 [21] 4 studies 1453 patients	Vasopressin vs. standard care	No effect on 28-day mortality Reduced need for RRT	Appears safe	

CCU, cardiac critical care unit; ICU, intensive care unit; RRT, renal replacement therapy; vs., versus.

## Data Availability

All the material used for this study would be available by the first author, on reasonable request.
